# Furin: A Potential Therapeutic Target for COVID-19

**DOI:** 10.1016/j.isci.2020.101642

**Published:** 2020-10-05

**Authors:** Canrong Wu, Mengzhu Zheng, Yueying Yang, Xiaoxia Gu, Kaiyin Yang, Mingxue Li, Yang Liu, Qingzhe Zhang, Peng Zhang, Yali Wang, Qiqi Wang, Yang Xu, Yirong Zhou, Yonghui Zhang, Lixia Chen, Hua Li

**Affiliations:** 1 Hubei Key Laboratory of Natural Medicinal Chemistry and Resource Evaluation, School of Pharmacy, Tongji-Rongcheng Center for Biomedicine, Tongji Medical College, Huazhong University of Science and Technology, Wuhan 430030, China; 2 Wuya College of Innovation, Key Laboratory of Structure-Based Drug Design & Discovery, Ministry of Education, Shenyang Pharmaceutical University, Shenyang 110016, China

**Keywords:** Computational Molecular Modelling, Disease, Drugs, Virology

## Abstract

COVID-19 broke out in the end of December 2019 and is still spreading rapidly, which has been listed as an international concerning public health emergency. We found that the Spike protein of SARS-CoV-2 contains a furin cleavage site, which did not exist in any other betacoronavirus subtype B. Based on a series of analysis, we speculate that the presence of a redundant furin cut site in its Spike protein is responsible for SARS-CoV-2's stronger infectious nature than other coronaviruses, which leads to higher membrane fusion efficiency. Subsequently, a library of 4,000 compounds including approved drugs and natural products was screened against furin through structure-based virtual screening and then assayed for their inhibitory effects on furin activity. Among them, an anti-parasitic drug, diminazene, showed the highest inhibition effects on furin with an IC_50_ of 5.42 ± 0.11 μM, which might be used for the treatment of COVID-19.

## Highlights

• The unique “RRAR” motif in SARS-CoV-2-S is easily recognized and hydrolyzed by furin
• A reason for high infectivity of SARS-CoV-2 is the furin cleavage site in Spike
• Furin is a potential therapeutic target for COVID-19
• Diminazene is a competitive inhibitor of furin, with an IC_50_ of 5.42 ± 0.11 μM

## Graphical Abstract

**Figure:**
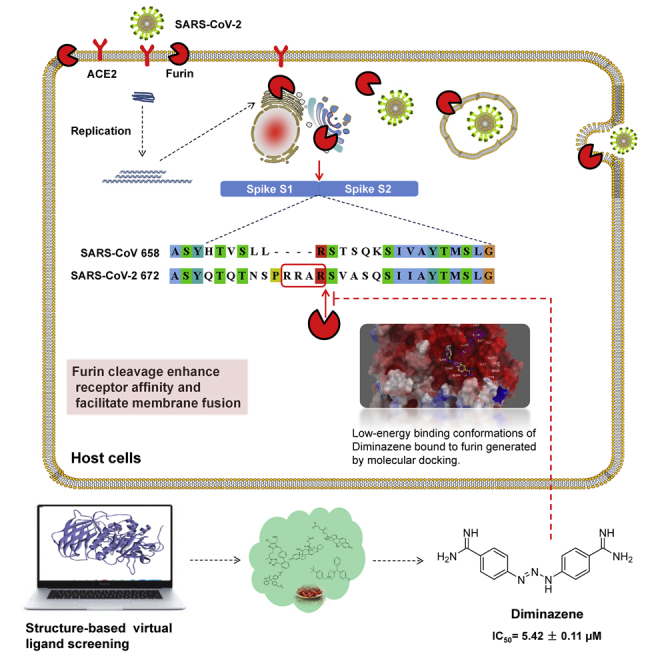

## Introduction

A novel coronavirus (SARS-CoV-2) infectious disease broke out in the end of December 2019 and is still spreading rapidly, which has been listed as an international concerning public health emergency. As of August 17, 2020, a total of 21,777,834 patients have been diagnosed and 776,544 have died worldwide. This disease is caused by a novel coronavirus, which was named “2019-nCoV” by the World Health Organization, and the disease caused by 2019-nCoV was named COVID-19. 2019-nCoV, as a close relative of SARS-CoV, was classified as severe acute respiratory syndrome coronavirus 2 (SARS-CoV-2) by the International Committee on Taxonomy of Viruses on February 11, 2020.

Coronaviruses (CoVs) are mainly composed of four structural proteins, including Spike (S), Membrane (M), Envelope (E), and Nucleocapsid (N) (Bosch et al., 2003). Spike, a trimeric glycoprotein of CoVs, determines the diversity of CoVs and host tropism and mediates CoVs' binding to host cells surface-specific receptors and virus-cell membrane fusion (Lu et al., 2015). Current research found that SARS-CoV-2 belongs to the β-coronavirus genus and speculated that it may interact with angiotensin-converting enzyme 2 (ACE2) on the surface of human cells through Spike protein, thereby infecting human respiratory epithelial cell (Xu et al., 2020). Letko and Munster then identified the receptor for SARS-CoV-2 entry into human cells to be ACE2 (Letko et al., 2020).

Coronavirus Spike protein plays a key role in the early stages of viral infection, with the S1 domain responsible for receptor binding and the S2 domain mediating membrane fusion (Belouzard et al., 2009). The process of SARS-CoV infecting the host involves two indispensable cleaving processes, which affect the infectious capacity of SARS-CoV. First, Spike was cleaved into receptor-bound N-terminal S1 subunit and membrane-fusion C-terminal S2 subunit by the host proteases at S1/S2 cleavage site (such as type II transmembrane serine protease [TMPRSS2], cathepsins B and L) (Glowacka et al., 2011; Zhou et al., 2015). Second, after CoVs are endocytosed by the host, the lysosomal protease mediates cleavage of S2 subunit (S2′ cleavage site) and releases the hydrophobic fusion peptide to fuse with the host cell membrane (Kam et al., 2009).

Furin, a kind of proprotein convertases (PCs), is located in the *trans*-Golgi network and activated by acid pH (Feliciangeli et al., 2006). Furin can cleave precursor proteins with specific motifs to produce mature proteins with biological activity. The first (P1) and fourth (P4) amino acids at the N terminus of the substrate cleavage site must be arginine “Arg-X-X-Arg ↓” (R-X-X-R, X: any amino acid, ↓: cleavage site). Kibler et al. demonstrated that the Spike protein S1/S2 and S2′ cleavage sites of the infectious bronchitis viruses (IBVs) Beaudette strain can be recognized by furin, which is a distinctive feature of IBV-Beaudette with other IBVs and has stronger infection ability (Tay et al., 2012; Yamada and Liu, 2009). Based on the characteristics of furin's recognition substrate sequence, some short peptide inhibitors have been developed, such as Decanoyl-Arg-Val-Lys-Arg-chloromethylketone (Dec-RVKR-CMK) and modified α1-antitrypsin Portland (α1-PDX). However, the non-specific and irreversible inhibitory effects on all members of the PC family limit their application (Henrich et al., 2003; Matsuyama et al., 2018). No small molecule inhibitor of furin with good effects and high specificity has been found so far.

The epidemiological observations showed that the infectious capacity of SARS-CoV-2 is stronger than that of SARS-CoV, so there are likely to be other mechanisms to make the infection of SARS-CoV-2 easier. We suppose the main possibilities as follows: first, SARS-CoV-2 RBD combining with ACE2 may have other conformations; second, the SARS-CoV-2 Spike protein can also bind to other receptors besides ACE2; third, Spike is more easily cleaved by host proteases and easily fused with host cell membrane. We compared the Spike proteins from three sources, SARS-CoV-2, SARS-CoV, and Bat-CoVRaTG13, and found that the SARS-CoV-2 virus sequence had redundant PRRA sequences. Through a series of analyses, this study proposes that one of the important reasons for the high infectivity of SARS-CoV-2 is a redundant furin cleavage site in its Spike protein. And through structure-based virtual ligand screening and *in vitro* enzyme-based assay, the anti-parasitic drug diminazene was found to show competitive inhibition on furin, with IC_50_ of 5.42 ± 0.11 μM.

## Results

### Bioinformatics Analysis Reveals Furin Cut Site in Spike Protein of SARS-CoV-2

**Figure 1:**
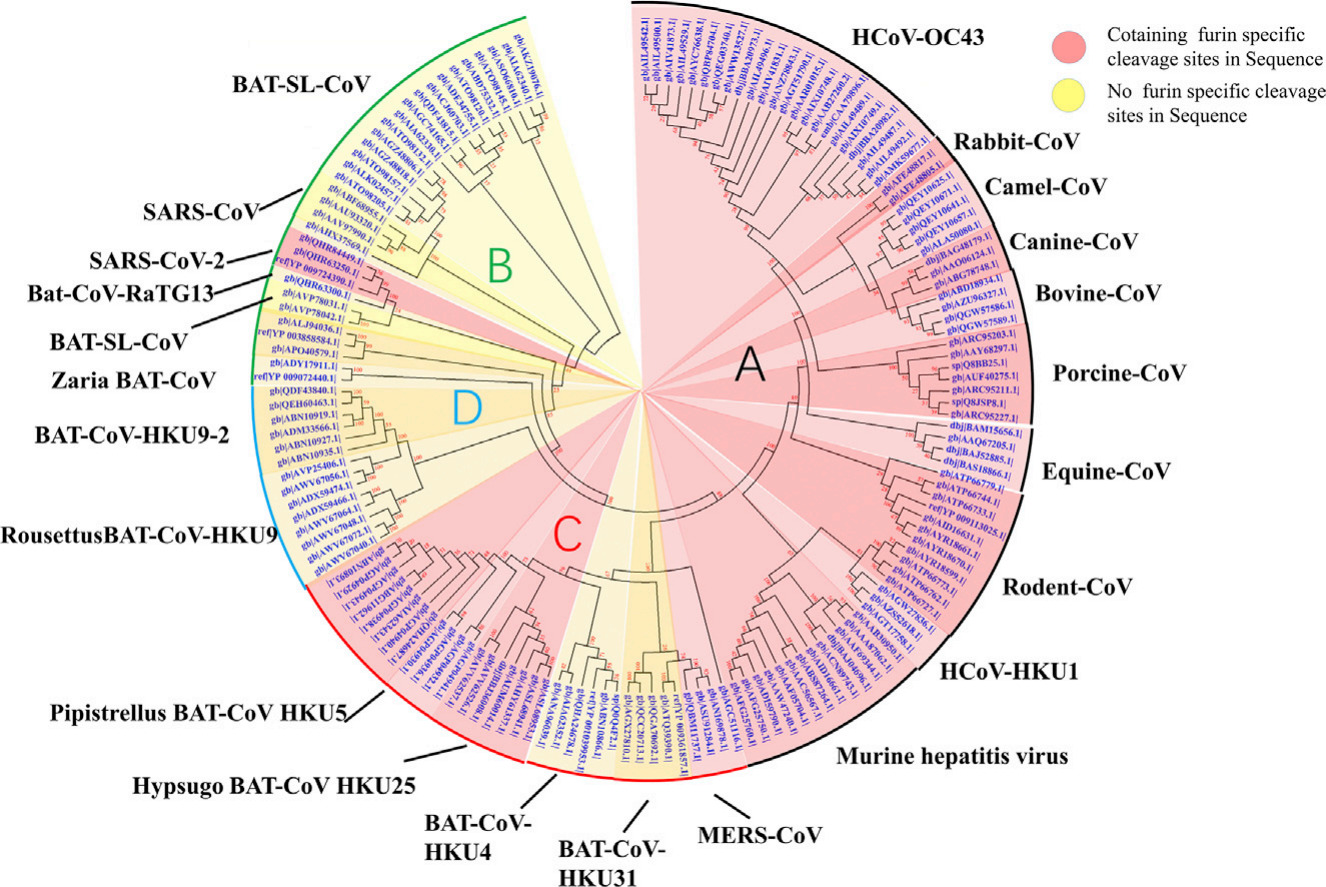
Evolutionary Relationships of Taxa The evolutionary history was inferred using the neighbor-joining method. The bootstrap consensus tree inferred from 500 replicates is taken to represent the evolutionary history of the taxa analyzed. Branches corresponding to partitions reproduced in less than 50% bootstrap replicates are collapsed. The evolutionary distances were computed using the Poisson correction method and in the units of the number of amino acid substitutions per site. The analysis involved 155 amino acid sequences. All positions containing gaps and missing data were eliminated. There are a total of 711 positions in the final dataset. Evolutionary analyses were conducted in MEGA7. Red shading means containing cleavage site in sequences and yellow shading means no cleavage site in sequences. All sequences are from β-coronavirus, and the four subtypes are marked in different outline colors.

By sequence alignment of Spike protein sequence of SARS-CoV-2 with its highly homologous sequences, it was found that the Spike cleavage site of SARS-CoV-2 possessed four redundant amino acids—PRRA, and these were not found in those of high-homology sequences, thus forming a furin-like restriction site as RRAR (Figures S1 and S2). Through prediction in ProP 1.0 Server, it is true that the sequence was easily digested by furin (Figure S3). To explore the evolution of this sequence, we used the BLASTp method to find 1,000 homologous Spike sequences with homology from 100% to 31%, which are all from β-CoVs. Multiple sequence alignments were performed on these thousands of Spike sequences. One sequence was selected from each highly homologous class (homology greater than 98.5%) for further sequence alignment, and about 155 sequences were finally selected. A homologous multiple sequence alignment was performed on these 155 sequences, and then a phylogenetic tree was constructed (Figure 1). As shown in the phylogenetic tree, the Spike of SARS-CoV-2 exhibited the closest linkage to those of Bat-SL-CoV and SARS-CoV, and far from those of MERS-CoV, HCoV-HKU1, and HCoV-OC43. In general, most of the Spike proteins in α-CoVs do not contain a furin cleavage site, but it is very popular in γ-CoVs' Spike protein, and with or without furin cleavage site are common in β-CoVs (Millet and Whittaker, 2015). We systematically analyzed the four subtypes of β-CoVs and found that only SARS-CoV-2 in the subtype B β-CoVs contains the furin cleavage site, and most of the subtype A β-coronaviruses contain the furin restriction site.

We performed furin digestion site prediction on the sequence of each type of coronavirus Spike through online software. It was found that all Spike with a SARS-CoV-2 Spike sequence homology greater than 40% did not possess a furin cleavage site (Figure 1, Table 1), including Bat-CoV RaTG13 and SARS-CoV (with sequence identity as 97.4% and 78.6%, respectively). The furin cleavage site “RRAR” of SARS-CoV-2 is unique in its family, rendering by its unique insert of “PRRA.” The furin cleavage site of SARS-CoV-2 is unlikely to have evolved from MERS, HCoV-HKU1, and so on. From the currently available sequences in databases, it is difficult for us to find the source. Perhaps there are still many evolutionary intermediate sequences waiting to be discovered.

**Table 1:** Furin Cleavage Probability of Spike Sequence Homology

Description	Accession No.	CS1 Sequence	Furin Score^a^	Identity^b^
SARS-CoV-2	QHR63250.1	NSPRRAR/SV	0.620	100%
Bat-CoV-RaTG13	QHR63300.1	QTQTNSR/SV	0.151	97.4%
Bat-SL-CoV	AVP78042.1	HTASILR/ST	0.170	80.3%
SARS-CoV	ABF68955.1	QLTPAWR/IY	0.117	76.0%
Bat-CoV HKU5	AGP04941.1	PSARLAR/SD	0.697	37.1%
MERS-CoV	QBM11737.1	LTPRSVR/SV	0.563	35.0%
Rat-CoV	AFG25760.1	TAHRARR/SV	0.879	36.3%
MHV	ABS87264.1	TSHRARR/SI	0.861	36.9%
HCoV-HKU1	AGT17758.1	SSRRKRR/GI	0.744	36.8%
Rodent-CoV	ATP66727.1	TARRKRR/AL	0.795	37.3%
β-CoV sp	AYR18670.1	ATRRAKR/DL	0.753	35.9%
Equine-CoV	BAS18866.1	TARRQRR/SP	0.815	37.1%
Porcine-CoV	ARC95227.1	TSLRSRR/SL	0.758	36.1%
Bovine-CoV	QGW57589.1	TKRRSRR/AI	0.780	37.5%
Canine-CoV	ABG78748.1	TQRRSRR/SI	0.832	37.1%
Camel-CoV HKU23	ALA50080.1	IDRRARR/FT	0.718	36.5%
Rabbit-CoV HKU14	AFE48805.1	TLQPSRR/AI	0.629	37.7%
Human-CoV OC43	AMK59677.1	KTRRSRR/AI	0.720	36.8%

a Scores are predicted by ProP 1.0 Server. Scores above 0.5 mean furin cleavable.

b Identities compared with SARS-CoV-2 Spike protein.

**Figure 2:**
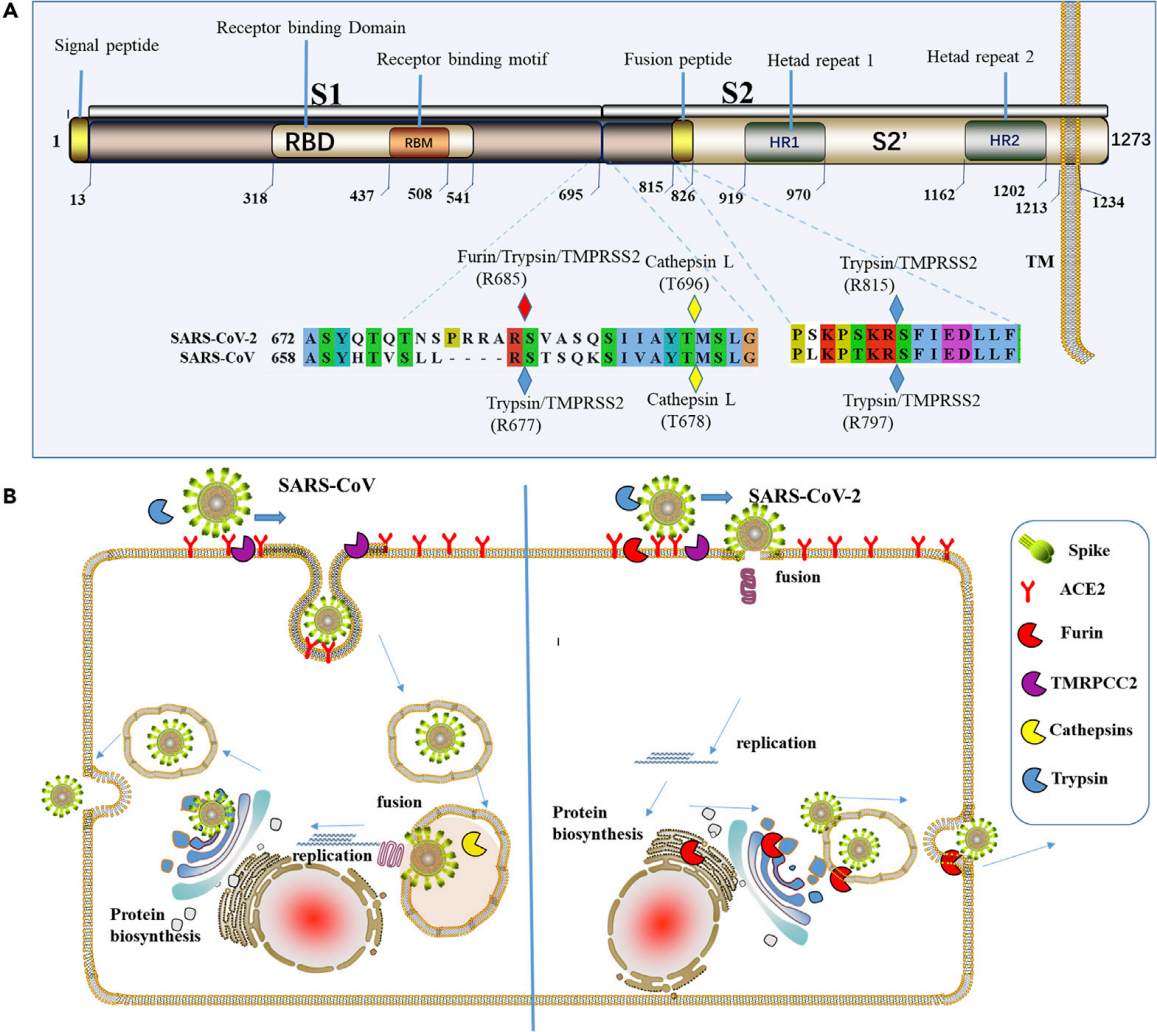
Analysis of SARS-CoV-2 Spike Protein and Its Membrane Fusion Mode (A) Sequence analysis of Spike protein in SARS-CoV-2. It contains an N-terminal signal peptide, S1 and S2. S1 contains N-terminal domain and receptor-binding region. S2 is mainly responsible for membrane fusion. The C-terminal region of S2 is S2′, and it contains a fusion peptide, HR1, HR2, and a transmembrane domain. The amino acid sequence numbers of every domain are annotated below them. Cleavage sites contained in SARS-CoV and SARS-CoV-2 are marked by rhombus. (B) A schematic diagram of the process of SARS-CoV and SARS-CoV-2-infecting host cells. Those proteases are presented by sector in different colors. Furin can cleave Spike in the process of viral maturation.

By analysis of the SARS-CoV-2 Spike protein sequence, we found that most of features are similar to those of SARS-CoV. It has an N-terminal signal peptide and is divided into two parts, S1 and S2. Among them, S1 contains N-terminal domain and receptor-binding region and S2 is mainly responsible for membrane fusion. The C-terminal region of S2 is S2′, containing a fusion peptide, heptad repeat 1, heptad repeat 2, and a transmembrane domain (Figure 2A). There are two cleavage sites between S1 and S2′, named CS1 and CS2. However, there are some differences in these two cleavage sites.

Unlike SARS-CoV, SARS-CoV-2 contains polybasic amino acids (RRAR) at the CS1 digestion site, and trypsin digestion efficiency will be significantly improved here (Belouzard et al., 2009). More importantly, as mentioned above, this site can be recognized and cleaved by the furin enzyme. The cleavage of Spike protein promotes structural rearrangements of RBD for the adaptation to receptor, thus increasing the affinity (Walls et al., 2019). More importantly, the digestion of Spike is indispensable for membrane fusion of S2 part (Kirchdoerfer et al., 2016). In this case, the cleavage efficiency of the SARS-CoV-2 Spike protein cleavage is significantly higher than that of SARS-CoV, and the SARS-CoV-2 Spike protein could be cleaved during the process of biosynthesis, which has been verified by a recent research (Walls et al., 2020) (Figure 2B). The receptor affinity and membrane fusion efficiency of SARS-CoV-2 would be significantly enhanced when compared with that of SARS-CoV. The membrane fusion of SARS-CoV-2 Spike protein is more likely to occur on the host cell plasma membrane. This may explain the strong infectious capacity of SARS-CoV-2. So, the development of furin inhibitors may be a promising approach to block its transmissibility.

### Homology Modeling and Protein-Protein Docking Calculation

In our previous studies (Wu et al., 2020), both SARS and SARS-CoV-2 Spike RBD structures have been docked with human ACE2 to calculate their binding free energy. In that time, the complex structure of SARS-CoV-2 RBD with ACE2 was not available. Its energy was calculated based on the homology model generated from SARS_RBD-ACE2 complex. The binding free energy between the SARS-CoV-2 Spike RBD and human ACE2 was −33.72 KCal mol^−1^ and that between SARS-CoV Spike RBD and ACE2 was −49.22 KCal mol^−1^. This means the binding affinity between SARS-CoV-2 Spike and ACE2 is weaker than that of SARS Spike. During this manuscript preparation, the structure of SARS-CoV-2 Spike RBD-ACE2 complex was disclosed (Wang et al., 2020). Based on this new real structure of SARS-CoV-2 Spike RBD-ACE2 complex, we re-did the calculation and found that the binding free energy between SARS-CoV-2 Spike RBD and ACE2 was −50.13 kcal mol^−1^ (Figure S4). This means the binding affinity between SARS-CoV-2 Spike and ACE2 is slightly stronger than that of SARS Spike. By inspecting the crystal structure of SARS-CoV-2 RBD-ACE2 complex and SARS RBD-ACE2 complex, one can find that one key loop of SARS-CoV-2 RBD in the complex interface had very different conformation compared with that of SARS RBD and previously modeled SARS-CoV-2 RBD (Figure S5).

To further explore the possible mechanism of furin cleaving SARS-CoV-2 Spike, we perform protein-protein docking for furin and Spike. We already built a homology model of SARS-CoV-2 Spike in our recently published article (Wu et al., 2020). SARS-CoV-2 Spike structure was built by using the SARS-CoV Spike structure as the template (PDB: 5X58) (Yuan et al., 2017). To verify the accuracy of homologous modeling, we aligned the computational structure of the SARS-CoV-2 Spike that modeled from the SARS-CoV spike with its cryoelectron microscopic (cryo-EM) structure (PDB: 6VXX) that was just solved and released when this manuscript was being revised and submitted (Walls et al., 2020). The computational model of the SARS-CoV-2 Spike showed a Cα root-mean-square deviation of 1.571 Å on the overall structure compared with the SARS-CoV-2 Spike cyro-EM structure, demonstrating a very subtle difference.

**Figure 3:**
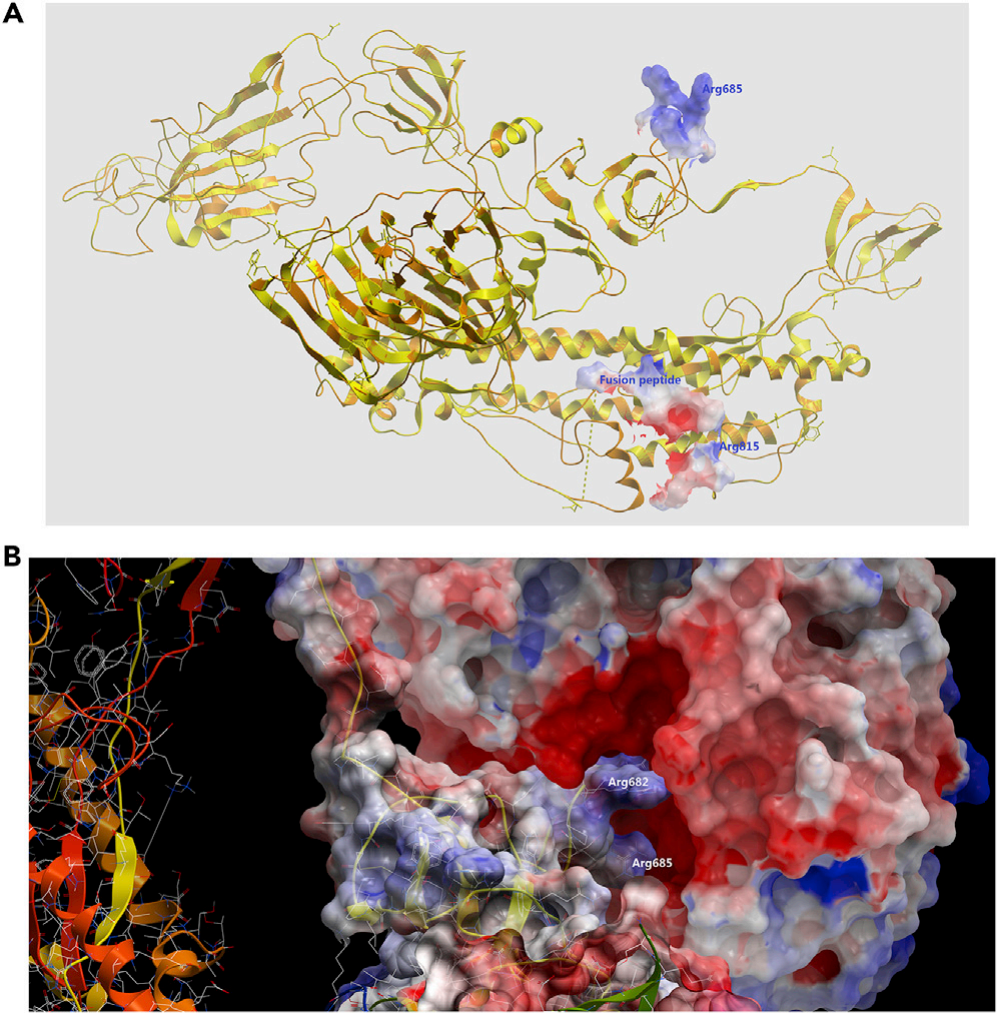
Protein-Protein Docking Model of SARS-CoV-2 Spike with Furin (A) Superimposition of SARS-CoV Spike and SARS-CoV-2 Spike. Two S1/S2 protease cleavage sites and fusion peptide were shown as electrostatic surface mode. (B) Furin was docked onto the putative furin cut site (Arg685) of SARS-CoV-2 Spike. Both domains are shown as electrostatic surface mode.

By superimposing the SARS-CoV Spike with the SARS-CoV-2 Spike, we can find that the major conformation differences between two structures are the RBD domain, Arg685/677 loop region (furin/trypsin/TMPRSS2 cut site), and S2 loop region just after fusion peptide (Figure 3A). The trypsin/TMPRSS2 cut sites of both SARS-CoV and SARS-CoV-2 Spikes were disordered and missing from the original cryo-EM structures possibly due to their flexibility and lack of electro density; we built this region by modeling software. The “PRRA” inserting in this region of SARS-CoV-2 apparently generates the more flexible loop region and accessible cut site for protease. We performed protein-protein docking by setting SARS-CoV-2 Spike furin cleavage loop as the receptor and furin active pocket as the ligand (Figure 3B). The protein-protein docking results showed that furin acidic/negative active pocket can be well fitted onto the SARS-CoV-2 Spike basic/positive S1/S2 protease cleavage loop with low energy (−18.43 KCal mol^−1^). This implies that the extra “PRRAR” loop of SARS-CoV-2 Spike renders it more fragile to the protease. And this may allow this site to be cut during the maturation, efficiently enhancing the infection efficiency.

### Validation of RRAR Motif as the Furin Cleavage Site

By the online ProP 1.0 Server software prediction, we proposed that furin can cleave the RRAR sequence, but the cleavage efficiency of this sequence has not been determined. To measure the cleavage efficiency, we performed a set of experiments to determine the Michaelis-Menten constant (Km) and limiting rate (Vmax) of furin using fluorogenic peptide substrate, Boc-RRAR-AMC (Table 2, Figure 7A). Kinetic parameters were also determined by using the second AMC substrate containing the authentic cleavage motif Arg-Val-Arg-Arg (Table 2, Figure 7A). Boc-RRAR-AMC has a similar Km value toward furin compared with Boc-RVRR-AMC. Using this substrate, a 2-fold higher hydrolysis efficiency was observed as demonstrated by the limiting rate, indicating that the “RRAR” motif is easily recognized by furin and quickly hydrolyzed.

**Table 2:** Kinetic Parameters of Furin with Different Substrates

Substrate	Km (μM)	Vmax (RFU/min)
Boc-RRAR-AMC	20.72 ± 1.30	221.5 ± 3.81
Boc-RVRR-AMC	19.16 ± 0.93	111.1 ± 1.45

### Virtual Ligand Screening of Furin Target and Validation

Structure-based virtual ligand screening method was used to screen potential furin protein inhibitors through ICM 3.7.3 modeling software (MolSoft LLC, San Diego, CA) from a ZINC Drug Database (2,924 compounds), a small in-house database of natural products (including reported common antiviral components from traditional Chinese medicine) and derivatives (1,066 compounds), and an antiviral compounds library containing 78 known antiviral drugs and reported antiviral compounds. Compounds with lower calculated binding energies (being expressed with scores and mfscores) are considered to have higher binding affinities with the target protein.

The screening results for the ZINC Drug Database (Table S1) showed that anti-tumor drugs aminopterin, fludarabine phosphate, and irinotecan; antibacterial drugs sulfoxone, lomefloxacin, and cefoperazone; antifungal drug hydroxystilbamidine; anti-parasitic drug diminazene; antiviral drug valganciclovir; hepatoprotective drug silybin; folic acid supplement folinic acid; etc., have higher binding affinity to furin with mfscores lower than −100 or scores lower than −30.

**Figure 4:**
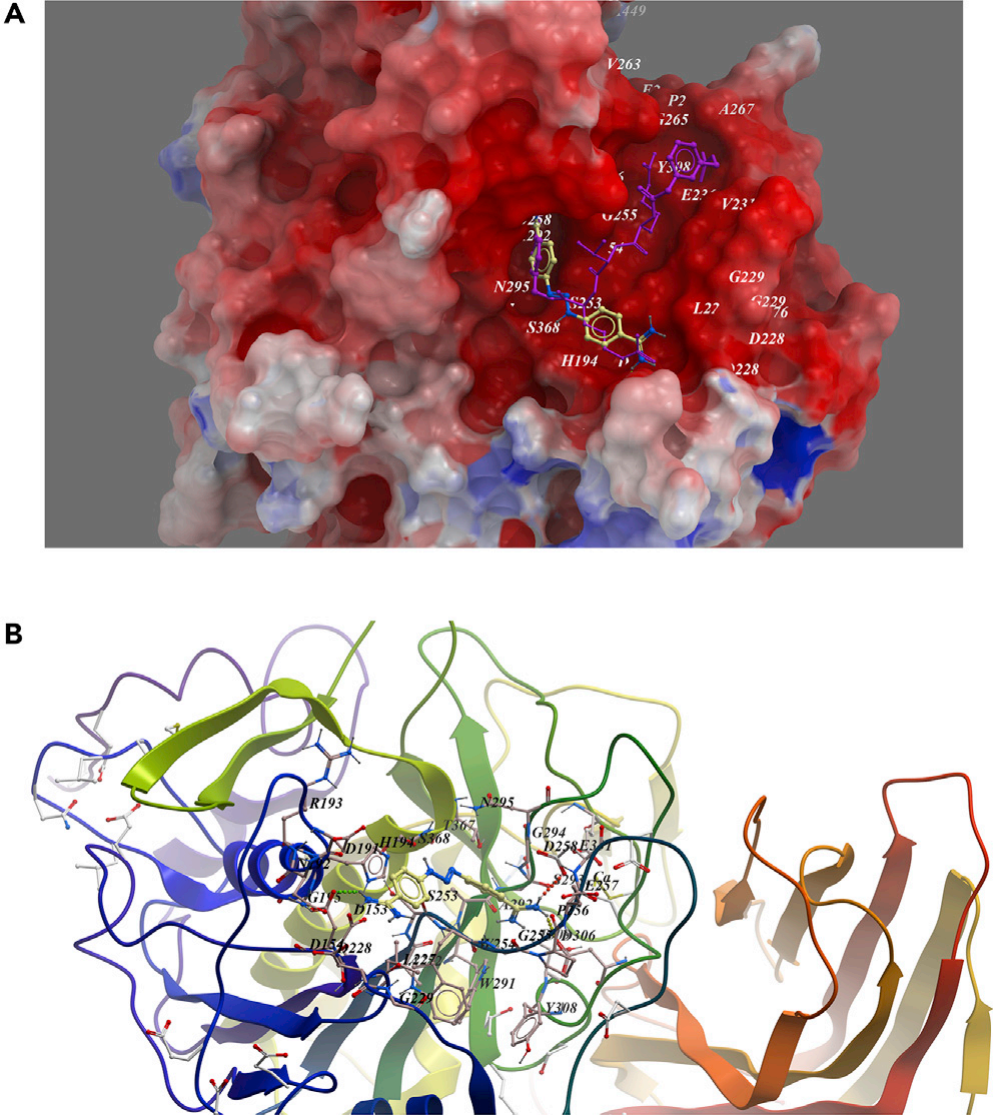
Low-Energy Binding Conformations of Diminazene Bound to Furin Generated by Molecular Docking (A) Diminazene was fitted well in the active pocket of human furin, and furin was shown as electrostatic surface model. Diminazene (yellow) was overlapped with substrate analog inhibitor MI-52 (purple). (B) Detailed view of diminazene binding in the active pocket of furin.

Here, we show one example of screen hits, diminazene, which was predicted to bind in the active site of furin with low binding free energy. In the generated docking model, diminazene was well fitted into the binding pocket of the substrate and adopted similar conformation as substrate analogous inhibitor MI-52 in PDB model PDB: 5JXH (Dahms et al., 2016), and occupied two arms' position of MI-52 (Figure 4A). Asp154, Asp258, and Asp306 were predicted to form three hydrogen bonds with imine groups of compounds (Figure 4B). It looks like that diminazene mimics at least two arginines. Weak hydrophobic interaction of His194, Leu227, the backbone of Trp254, and Asn295 with the compound may further stabilize its conformation.

**Figure 5:**
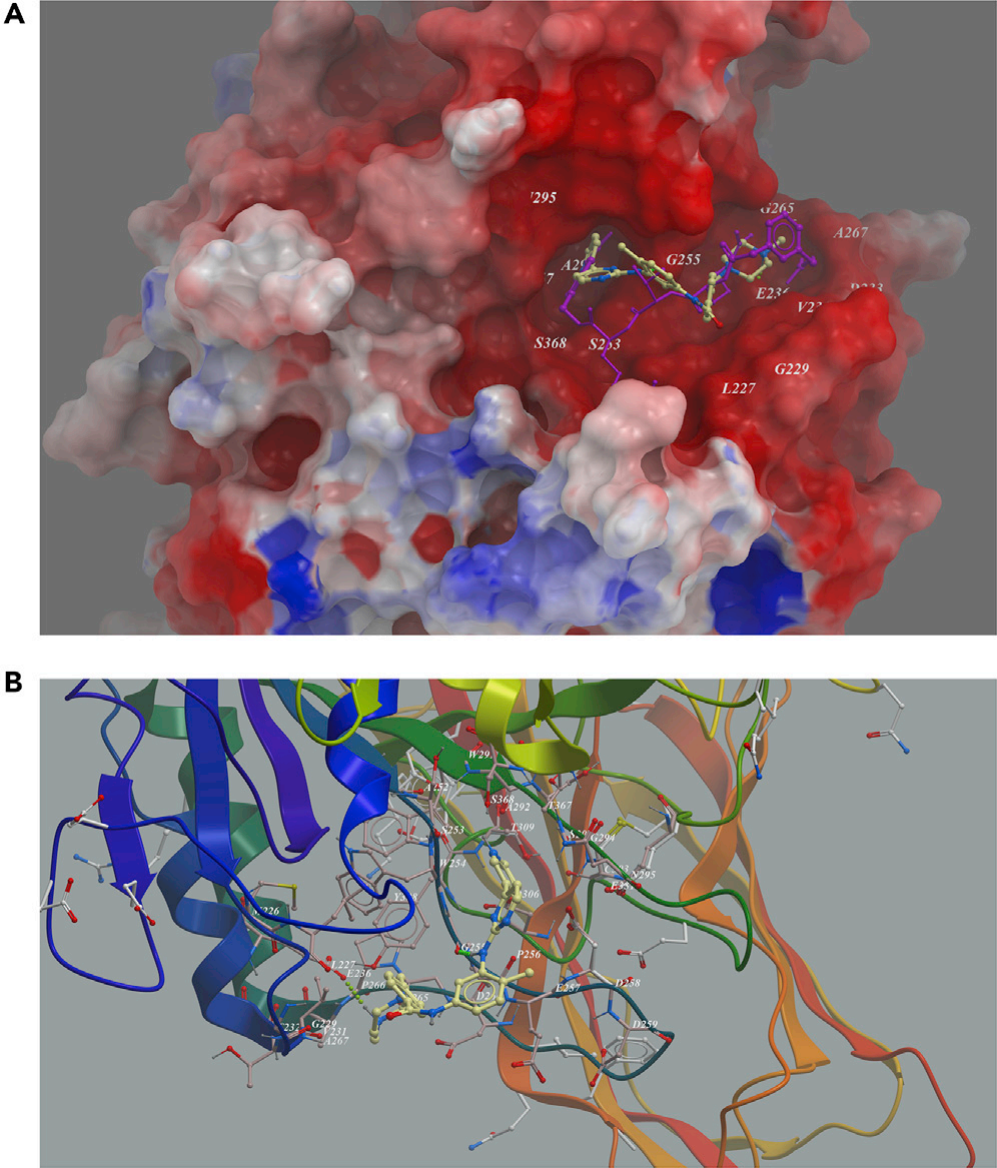
Low-Energy Binding Conformations of Imatinib to Furin Generated by Molecular Docking (A) Imatinib was fitted well in the active pocket of human furin, and furin was shown as electrostatic surface model. Imatinib (yellow) was overlapped with substrate analog inhibitor MI-52 (purple). (B) Detailed view of Imatinib binding in the active pocket of furin.

Another example was anticancer drug imatinib. It was also predicted to bind in the active site of furin. In the generated docking model, imatinib was fitted well in the binding pocket and occupied the top two arms' position of MI-52 (Figure 5A). Two hydrogen bonds were predicted to form between the compound with Glu236 and Gly255. Weak hydrophobic interaction between Val231, Pro256, Trp254, and Gly294 and the compound was found (Figure 5B).

For the natural products (Table S2), a series of compounds with antiviral and anti-inflammation effects, such as (−)-epigallocatechin gallate (ECCG) and theaflavin 3,3′-di-*O*-gallate from *Camellia sinensis*, biorobin from *Ficus benjamina*, andrographolide and 14-deoxy-11,12-didehydroandrographiside from *Andrographis paniculata*, 2*β*,30*β*-dihydroxy-3,4-seco-friedelolactone-27-lactone from *Viola diffusa*, phyllaemblicin G7 from *Phyllanthus emblica*, and xanthones kouitchenside J and kouitchenside F from *Swertia kouitchensis* exhibited potential high binding affinity to furin protein (mfscores < −100).

**Figure 6:**
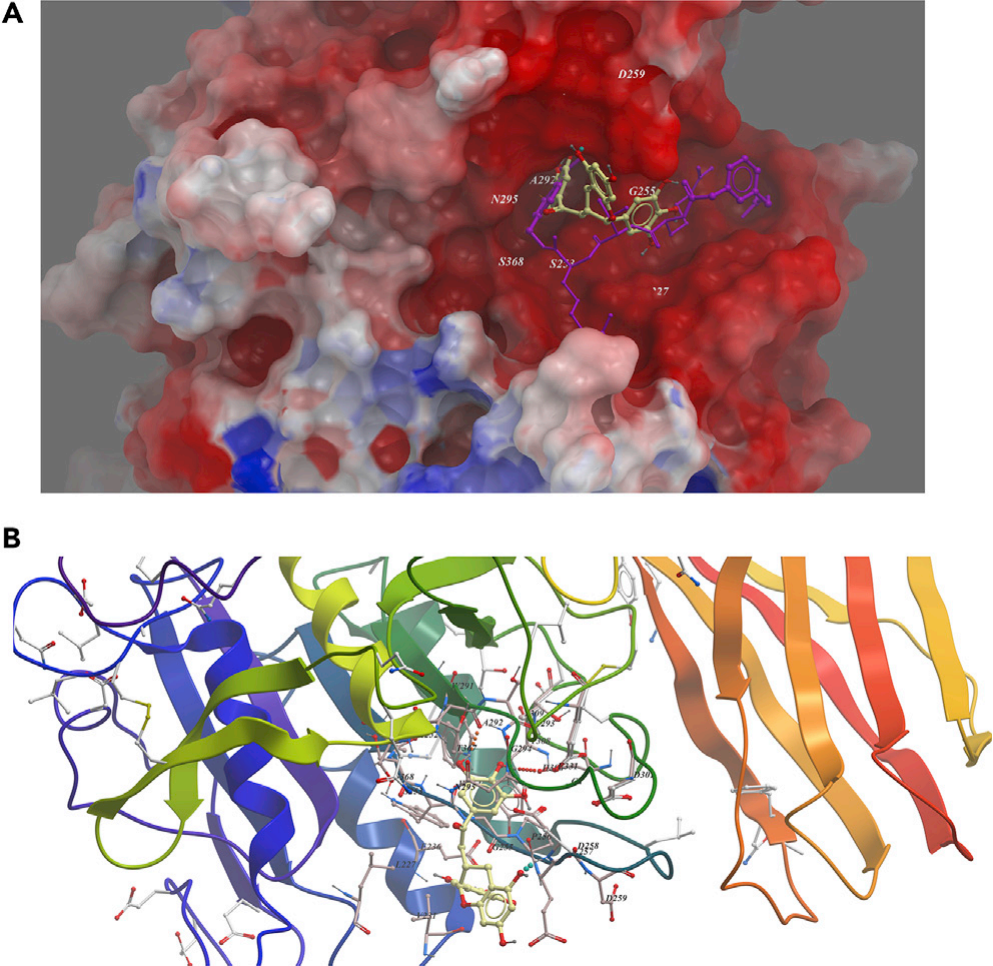
Low-Energy Binding Conformations of ECCG to Furin Generated by Molecular Docking (A) ECCG was fitted well in the active pocket of human furin, and furin was shown as electrostatic surface model. ECCG (yellow) was overlapped with substrate analog inhibitor MI-52 (purple). (B) Detailed view of ECCG binding in the active pocket of furin.

EGCG was predicted to bind in the active site of furin, as imatinib; it occupied the top two arms' position of MI-52 (Figure 6A). Two hydrogen bonds were predicted to be formed between the compound with Asp258 and Ala292. Weak hydrophobic interactions between Pro256, Trp254, and Gly294 and the compound were predicted (Figure 6B).

The database of 78 antiviral drugs including compounds already on the market and currently undergoing clinical trials to treat SARS-CoV-2 infections was further screened. The results were shown in Table S3. DNA topoisomerase II inhibitor suramin treating hand-foot-and-mouth disease exhibited the highest affinity with furin (mfscore = −190.406). Indinavir, tenofovir alafenamide, tenofovir disoproxil, isoproxil, dolutegravir, boceprevir, and telaprevir may also have high binding affinity with furin.

**Figure 7:**
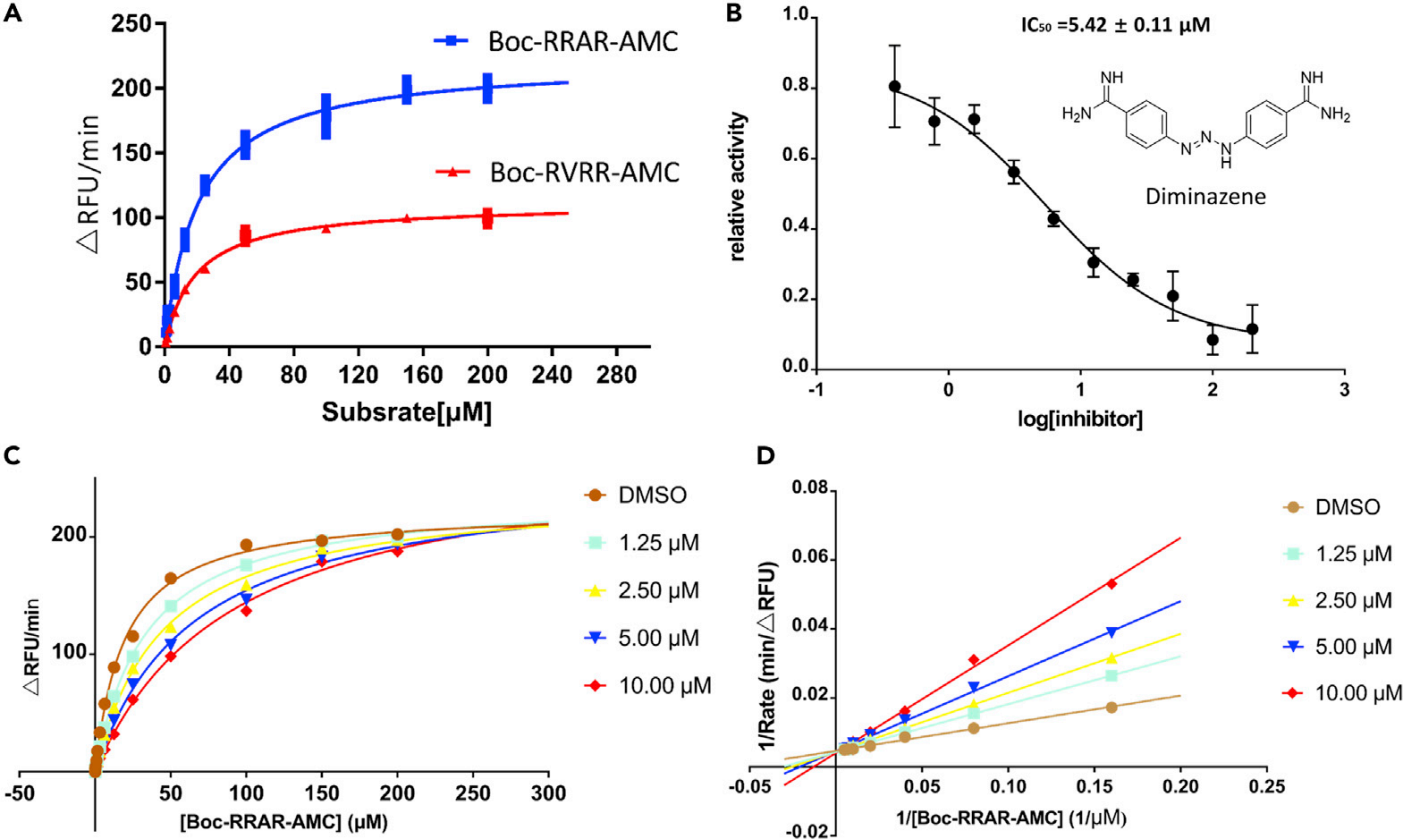
*In Vitro* Enzymatic Characterization of Recombinant Human Furin Protease and Enzyme Inhibition Assay (A) Substrate saturation profiles for furin. Representative plots of the initial rate (Vi in ▵RFU/min) versus the amounts of fluorogenic substrate Boc-RVRR-AMC or Boc-RRAR-AMC. The *Vmax* and *Km* were determined by fitting the experimental data to a pseudo-first-order rate curve yielding the computed value and the best fit curve. (B) Dose response inhibition of furin activity by diminazene. (C) Substrate saturation profiles for furin at a range of concentrations of diminazene. (D) Lineweaver-Burk double-reciprocal representation of the Boc-RRAR-AMC saturation profiles for furin at a range of concentrations of Boc-RRAR-AMC.

To validate the finding of virtual ligand screening, the recombinant human furin was expressed in HEK-293-GnTI and purified. We tested the furin enzyme inhibitory effect of all the available compounds with a final concentration of 100 μM (Table S4). Among them, the anti-parasitic drug diminazene showed an inhibition ratio over 95%, and other compounds like aminopterin, methotrexate, and silybin showed inhibition ratio over 70%. Among them, diminazene showed significantly dose-dependent inhibition on furin protease, with IC_50_ value of 5.42 ± 0.11 μM (Figure 7B). Enzyme kinetics has also been measured to explore its mode of inhibition. As shown in Figures 7C and 7D, diminazene displayed a competitive inhibition mechanism characterized by dose-dependent increase in Km and little effects on Vmax (Table 3).

**Table 3:** Kinetic Parameters of Furin with Different Concentrations of Diminazene

Concentration of Diminazene (μM)	Km (μM)	Vmax (RFU/min)
DMSO	19.84 ± 1.02	226.0 ± 2.8
1.25	33.28 ± 0.84	234.7 ± 1.6
2.5	43.09 ± 2.25	236.7 ± 3.7
5.0	61.86 ± 4.02	248.9 ± 5.5
10.0	76.31 ± 7.32	252.1 ± 9.0

## Discussion

Our previous study (Wu et al., 2020) analyzed the amino acid composition of the RBD domain of the ACE2 receptor of SARS-CoV-2 and Bat-CoVRaTG13. We found that several key amino acids determining binding were mutated in SARS-CoV-2, which are more similar to that of SARS-CoV. The calculation results showed that in the same conformation as the SARS-CoV protein, the binding free energy of SARS-CoV-2 and ACE2 receptors was a little higher, but this result cannot fully explain the epidemiologically high contagion, so we speculate that (1) the RBD domain of SARS-CoV-2 may have other conformations, (2) there may be other receptors, and (3) there are other mechanisms that enhance infectivity. During the preparation of this manuscript, the cryo-EM structure of SARS-CoV-2 Spike was solved (Walls et al., 2020). Comparing the structure of SARS-CoV-2 with the Spike structure of SARS-CoV, combined with biophysical detection, they found that SARS-CoV-2 binds more strongly to cellular ACE2 receptors (Walls et al., 2020). Furthermore, the just disclosed crystal structure of SARS-CoV-2 RBD-ACE2 complex showed a distinct conformational change in the key loop of complex binding interface. And the binding free energy calculation indicated a slightly stronger binding for SARS-CoV-2 RBD compared with that for SARS RBD. These results confirm our supposition that the conformational change of the RBD domain of SARS-CoV-2 leads to stronger binding. However, stronger receptor binding still cannot fully explain the more infectious problem of SARS-CoV-2.

So we put forward the following hypotheses: (1) SARS-CoV-2 can also bind to other receptors, (2) the lung may not be the earliest infection site, and (3) SARS-CoV-2 is easier to be cut and more easily fuses with cell membranes. As published in the Pubmed database, researchers performed RNA sequencing analysis on tissue samples from 95 individuals' 27 different tissues. The results showed that ACE2 protein was highly expressed in the small intestine and duodenum, but the expression level in lung tissue is low (Figure S6). However, we analyzed the expression of furin and found that it is distributed in various organs with little difference in expression level. Combined with the possible infection mechanism of SARS-CoV-2, the widespread distribution of furin increases the SARS-CoV-2 infection of other organs. The possibility of other organ attack is consistent with the multiple symptoms observed in clinic of COVID-19.

Based on these three conjectures, we compared the Spike sequences from SARS-CoV-2, SARS-CoV, and Bat-CoVRaTG13 and found an extra “PRRA” insert near the S1/S2 cleavage site. During the preparation of this manuscript, there were also a few studies reporting this potential furin site. Xin Li et al. proposed the possible furin cleavage sites (Li et al., 2020), but their classification of β-coronavirus was unclear, and their speculation that the packaging mechanism of SARS-CoV-2 was similar to HIV and Ebola viruses was insufficiently supported. This site was also reported in another article and aligned with several other human coronaviruses (Coutard et al., 2020). In our research, furin score of this site predicted by ProP 1.0 server was 0.62, and the feasibility of furin cutting in this site of the Spike protein of SARS-CoV-2 was evaluated by protein-protein docking and free energy calculation. More importantly, we simulated the recognition and hydrolysis of RRAR sequence by furin through hydrolysis of the fluorescent substrate Boc-RRAR-AMC and found that the substrate Boc-RRAR-AMC can be effectively recognized and cleaved by furin, and its hydrolysis efficiency was higher than Boc-RVRR-AMC, a known substrate of furin.

We systematically analyzed the four subtypes of β-CoV and found that SARS-CoV-2 was the only one in the subtype B β-CoV that contains the furin cleavage site, whereas most of the subtype A contains the furin restriction site (Figure 1). We aligned 1,000 Spike sequences and found that all Spikes with sequence homology greater than 40% of SARS-CoV-2 Spike did not have a furin cleavage site, but its possible evolutionary source cannot be found currently, and more novel viruses are needed to be discovered. Very recently, a new closely related virus RmYN02 containing PAA at the CS1 of the S protein has been reported, but it also had no furin cleavage sites (Zhou et al., 2020).

The “PRRA” insert and subsequent arginine (R) constitute an RRAR sequence that could be recognized and cleaved by furin-like proteases, which may be the reason why SARS-CoV-2 infection is stronger than SARS-CoV. What's more, we performed a homologous alignment and phylogenetic analysis of the SARS-CoV-2 sequence and found that “PRRA” insert did not appear in any other close relatives of SARS-CoV-2, indicating that this insert was completely novel in this genus of virus. The existence of such a motif may allow Spikes to be cut into S1 and S2 by furin-like proteases before maturity, which provides S1 with the flexibility to change the conformation to better fit the host receptor. According to studies of Simmons et al., overexpression of furin can increase the activity of SARS-CoV Spike, but it will not cause Spike to be cleaved (Simmons et al., 2011). This is consistent with our prediction.

Furthermore, Glowacka et al. and Simmons et al. have demonstrated that SARS-CoV Spikes can be activated by cleavage in two ways, including proteolytic activation by cathepsins B and L in host cells (Simmons et al., 2005). In addition, SARS-CoV Spike can be activated by TMPRSS2 cleavage on the host cell surface (Glowacka et al., 2011). As we can see in Figure 2B, the Spike protein of SARS-CoV-2 can be cleaved at multiple stages, which greatly increases the efficiency of fusion. Markus et al. demonstrated that the CS1 of the S protein of SARS-CoV-2 was easily cleaved in the host cell, and they mentioned that TMPRSS2 plays an important role in the cleavage of CS2 (Hoffmann et al., 2020). It is likely that the virus will fuse with the cell in the cell plasma membrane and release the genome into cells. In addition, the receptor affinity of the cleaved Spike is also greatly enhanced (Parka et al., 2016).

According to our study, furin-like proteases may be potential drug targets for anti-SARS-CoV-2 treatment. At present, some peptide inhibitors have been developed and have good effects (Dahms et al., 2018; Dahms et al., 2017). To search for potential inhibitors of furin-like proteases, we screened potential compounds from a ZINC drug database (2,924 compounds), a small in-house database of natural products (1,066 compounds), and existing antiviral drugs library (78 compounds) with furin by virtual ligand screening. We found that a series of anti-tumor, antibacterial, antiviral, anti-parasitic, and hepatoprotective drugs may have high binding affinity to furin.

To validate the results of the virtual ligand screening, we tested the furin enzyme inhibitory effect of all the available compounds with a final concentration of 100 μM. It was found that some of these compounds did exhibit certain inhibitory effects on furin, such as aminopterin, silybin, diminazene, methotrexate, lomefloxacin, imatinib, tenofovir, disoproxil, etc. Among them, diminazene showed the strongest inhibitory activity with an IC_50_ of 5.42 ± 0.11 μM. It was found to be a competitive inhibitor by analyzing its enzyme kinetics characteristic on furin. Diminazene is probably occupied the substrate-binding pocket of furin, which is consistent with the result of molecular docking. Diminazene might be a drug candidate for the treatment of COVID-19 by inhibiting furin, or can at least serve as a lead compound to guide the development of novel furin inhibitors for the treatment of COVID-19. What's more, combined administration of furin inhibitors targeting different SARS-CoV-2 proteases may be an effective therapeutic strategy. The furin inhibitor here might open a new avenue for the treatment of COVID-19. Further experiments to verify its efficiency both *in vitro* and *in vivo* will be carried out in our future studies.

### Limitation of the Study

A total of 1,000 SARS-CoV-2 Spike homologous sequences were searched through BLASTp method, but the Spike proteins of the newly discovered viruses, such as RmYN02 and pangolin-CoV, were not included in the database. So several Spike protein sequences were not analyzed in this study.

### Resource Availability

#### Lead Contact

Further information and requests for resources should be directed to the Lead Contact, Hua Li (li_hua@hust.edu.cn).

#### Materials Availability

Materials and the information used for the experiments are available upon reasonable request.

#### Data and Code Availability

All data used in the study are included in this publication. The present research did not use any new codes.

## Methods

All methods can be found in the accompanying Transparent Methods supplemental file.

## Supplemental Information

Document S1. Transparent Methods, Figures S1–S6, and Tables S1–S4

Data S1. Generated Docking Model of Furin-Spike Complex, Related to Figure 3
