# Supplementary material for: Furin: A Potential Therapeutic Target for COVID-19
Source: iScience. 2020 Oct 5;23(10):101642. doi: 10.1016/j.isci.2020.101642 (PMC7534598; doi:10.1016/j.isci.2020.101642)
Supplement: Document S1. Transparent Methods, Figures S1–S6, and Tables S1–S4 [file mmc1.pdf]

iScience, Volume *23*

Supplemental Information

Furin: A Potential Therapeutic

Target for COVID-19

Canrong Wu, Mengzhu Zheng, Yueying Yang, Xiaoxia Gu, Kaiyin Yang, Mingxue Li, Yang Liu, Qingzhe Zhang, Peng Zhang, Yali Wang, Qiqi Wang, Yang Xu, Yirong Zhou, Yonghui Zhang, Lixia Chen, and Hua Li

Supplementary information

Furin, a potential therapeutic target for COVID-19

Canrong Wu,[a,1] Mengzhu Zheng,[a,1] Yueying Yang,[b,1] Xiaoxia Gu,[a] Kaiyin Yang,[a] Mingxue Li,[b] Yang Liu,[b] Qingzhe Zhang,[a] Peng Zhang,[b] Yali Wang,[b] Qiqi Wang, [b] Yang Xu,[b] Yirong Zhou,[a,*] Yonghui Zhang,[a,*] Lixia Chen,[b,*] Hua Li[a,b,*,2]

[a]*Hubei Key Laboratory of Natural Medicinal Chemistry and Resource Evaluation, School of Pharmacy, Tongji-Rongcheng Center for Biomedicine, Tongji Medical College, Huazhong University of Science and Technology, Wuhan 430030, China*
[b]*Wuya College of Innovation, Key Laboratory of Structure-Based Drug Design & Discovery, Ministry of Education, Shenyang Pharmaceutical University, Shenyang 110016, China*

[1] These authors contributed equally to this work.
[2]Lead Contact.

*Corresponding author: Hua Li, Lixia Chen, Yonghui Zhang & Yirong Zhou
E-mail: li_hua@hust.edu.cn (H. Li)
syzyclx@163.com (L. Chen)
zhangyh@mails.tjmu.edu.cn (Y. Zhang)
zhouyirong@hust.edu.cn (Y. Zhou),

Transparent Methods

Homology Spike protein blast and sequence alignment.

The Spike protein of (GB: QHR63250.1) was downloaded from NCBI nucleotide database. The protein sequence were aligned with whole database using BLASTp to search for homology viral Spike protein (Alogorithm parameters, Max target sequences: 1000, Expect threshold: 10). Multiple-sequence alignment was conducted in BLASTp online and analysis with DNAMAN and Jalview. The evolutionary history was inferred using the Neighbor-Joining method in MEGA 7 software package. The percentage of replicate trees in which the associated taxa clustered together in the bootstrap test was determined by 500 replicates. The Spike protein sequence analyses were conducted in snapgene view.

Furin cleavage site prediction

The prediction of furin cleavage sites were carried out in ProP 1.0 Server (http://www.cbs.dtu.dk/services/ProP/).

Compounds database

Approved drug database was from the subset of ZINC database, ZDD (ZINC drug database) containing 2924 compounds (Irwin et al., 2012). Natural products database was constructed by ourselves, containing 1066 chemicals separated from traditional Chinese herbals in own lab and natural-occurring potential antiviral components and derivatives. Antiviral compounds library contains 78 known antiviral drugs and reported antiviral compounds through literature search.

Homology modeling and molecular docking

Corresponding homology models predicted by Fold and Function Assignment System server for each target protein were downloaded from Protein Data Bank (www.rcsb.org). Alignment of two protein sequences and subsequent homology modeling were performed by bioinformatics module of ICM 3.7.3 modeling software on an Intel i7 4960 processor (MolSoft LLC, San Diego, CA). For the structure-based virtual screening, ligands were continuously resiliently made to dock with the target that was represented in potential energy maps by ICM 3.7.3 software, to identify possible drug candidates. 3D

compounds of each database were scored according to the internal coordinate mechanics (Internal Coordinate Mechanics, ICM) (Abagyan et al., 1994). Based on Monte Carlo method, stochastic global optimization procedure and pseudo-Brownian positional/torsional steps, the position of intrinsic molecular was optimized. By visually inspecting, compounds outside the active site, as well as those weakly fitting to the active site were eliminated. The software adopted two kind of scoring system. One is ICM score, which is based on the empirical function of predicted physical interaction, calculated according to seven parameters, including ligand-target hydrogen bonding interactions,internal force-field energy of the ligand, desolvation energy of hydrogen bond donor-acceptor,entropy loss due to conformational differences upon ligand binding, polar and non-polar solvation energychanges upon ligand binding,electrostatic energyand hydrophobic free energy (Neves et al., 2012). Another is mfscore, it is a potential of mean force score and provides an independent score of the strength of ligand-receptor interaction. It is a measure of statistical probability of interaction between the ligand and the receptor. It examines interatomic distances of the docked interaction, and compares that to existing interactions available in PDB (Muegge et al., 1999).

Compounds with Scores less than -30 or mfScores less than -100 (generally represents strong interactions) have priority to be selected. Protein-protein docking procedure was performed according to the ICM-Pro manual.

Expression and purification of furin

The detailed procedures for expression and purification of human furin (UNIPROT ID P09958) refer to previous work with some modifications (Dahms et al., 2018). Briefly, the sequence encoding human furin amino acids 23-574 was cloned into PEGMan vector with N-terminal secretion signal peptide and C-terminal His tag. The expressed plasmid was transfected in HEK293-GnTI cells using polyethylenimine transfection reagent, and the transfected cells were cultured in a 10 cm plate with 10 mL of DMEM (Invitrogen) containing 10% bovine calf serum (HyClone, GE Healthcare) overnight. The medium was changed to 10 mL of freeStyle 293 Expression Media (Thermo Fisher Scientific), and the

cells were cultured for another 72 h. The conditioned medium was collected and centrifuged at 4500 g for 20 min. The supernate protein was dialysis against 25 mM Tris, pH 8.0, 250 mM NaCl, 5 mM $CaCl_2$ overnight at 20 °C before purification. The secreted furin was purified from the culture media using Ni-NTA agarose (Qiagen) affinity chromatography with gravity flow.

Enzymatic activity and inhibition assays

In brief, the activity of human furin was measured by a continuous kinetic assay, with the substrate Boc-Arg-Arg-Ala-Arg-AMC (GL Biochem) or Boc-Arg-Val-Arg-Arg-AMC (GL Biochem), using wavelengths of 360±40 nm and 460±40 nm for excitation and emission, respectively. Furin was added in assay buffer containing 100 mM HEPES buffer, pH 7.0, 0.2% (v/v) Triton X-100, 2 mM $CaCl_2$ and 0.1 mg/mL BSA. The assay started by immediately mixing with different concentrations of substrate (0.39-200 μM). The fluorophore, 7-amino-4-methylcoumarin (AMC) group released from Boc-RRAR-AMC or Boc-RVRR-AMC was monitored in the form of relative fluorescence units as a function of time (RFU/min) using a BioTEK Synergy H1 multimode microplate reader at 37 °C. The IC_{50} values were calculated by fitted regression equation using the-log plot (GraphPad Prism). Each value was expressed with the means ± SD of three independent tests, each with three replicates.

Supplemental References

Abagyan, R., Totrov, M., Kuznetsov, D. (1994). ICM-a new method for protein modeling and design-applications to docking and structure prediction from the distorted native conformation. J Comput Chem *15*, 488-506.

Dahms, S.O., Hardes, K., Steinmetzer, T., Than, M.E. (2018). X-ray Structures of the proprotein convertase furin bound with substrate analogue inhibitors reveal substrate specificity determinants beyond the S4 pocket. Biochemistry *57*, 925-934.

Irwin, J.J., Sterling, T., Mysinger, M.M., Bolstad, E.S., Coleman, R.G. (2012). ZINC: a free tool to discover chemistry for biology. J Chem Inf Model *52*, 1757-1768.

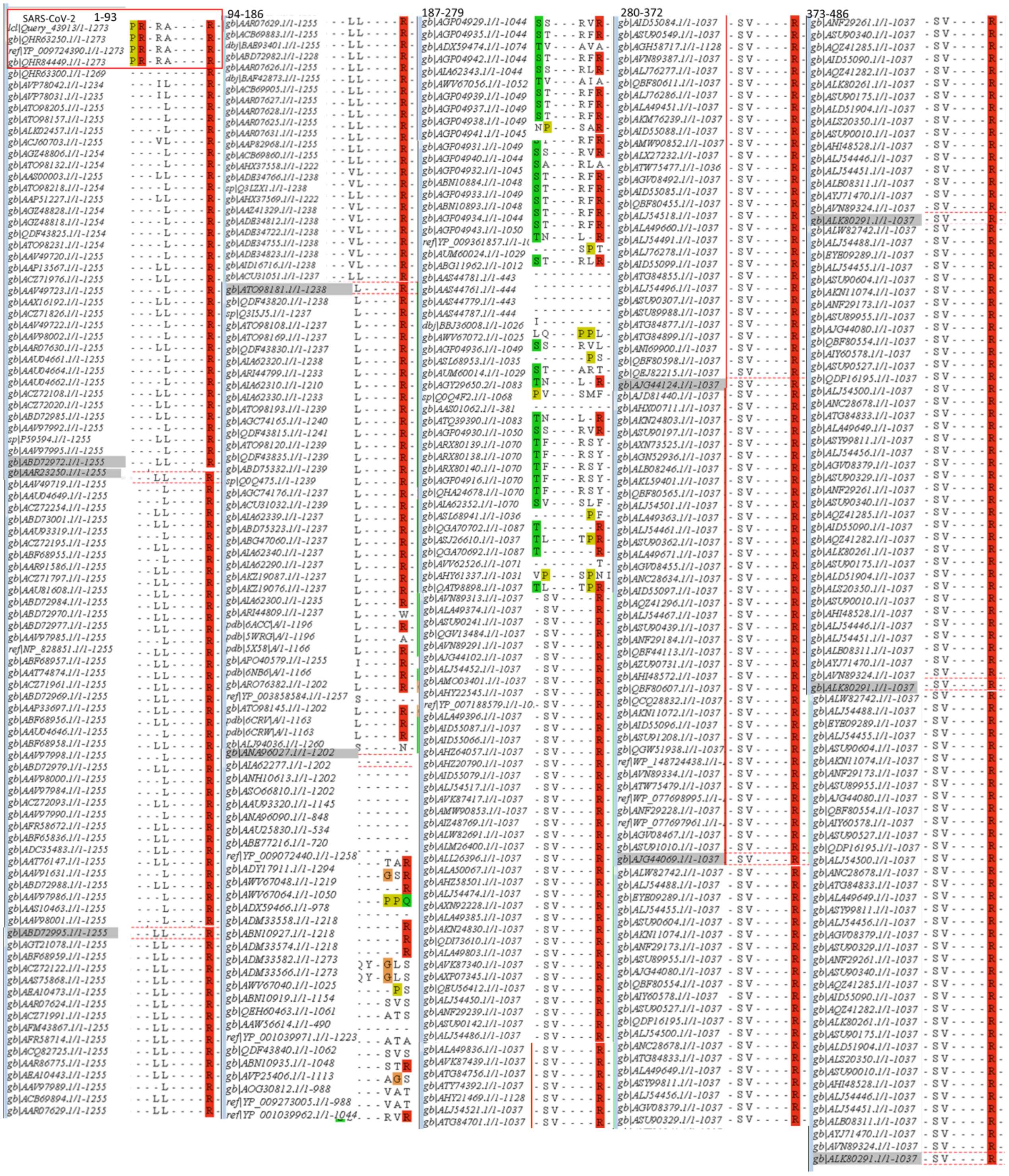

Figure S1. Multiple sequence alignment of 1000 Spike proteins(1-486). Related to Figure 1.

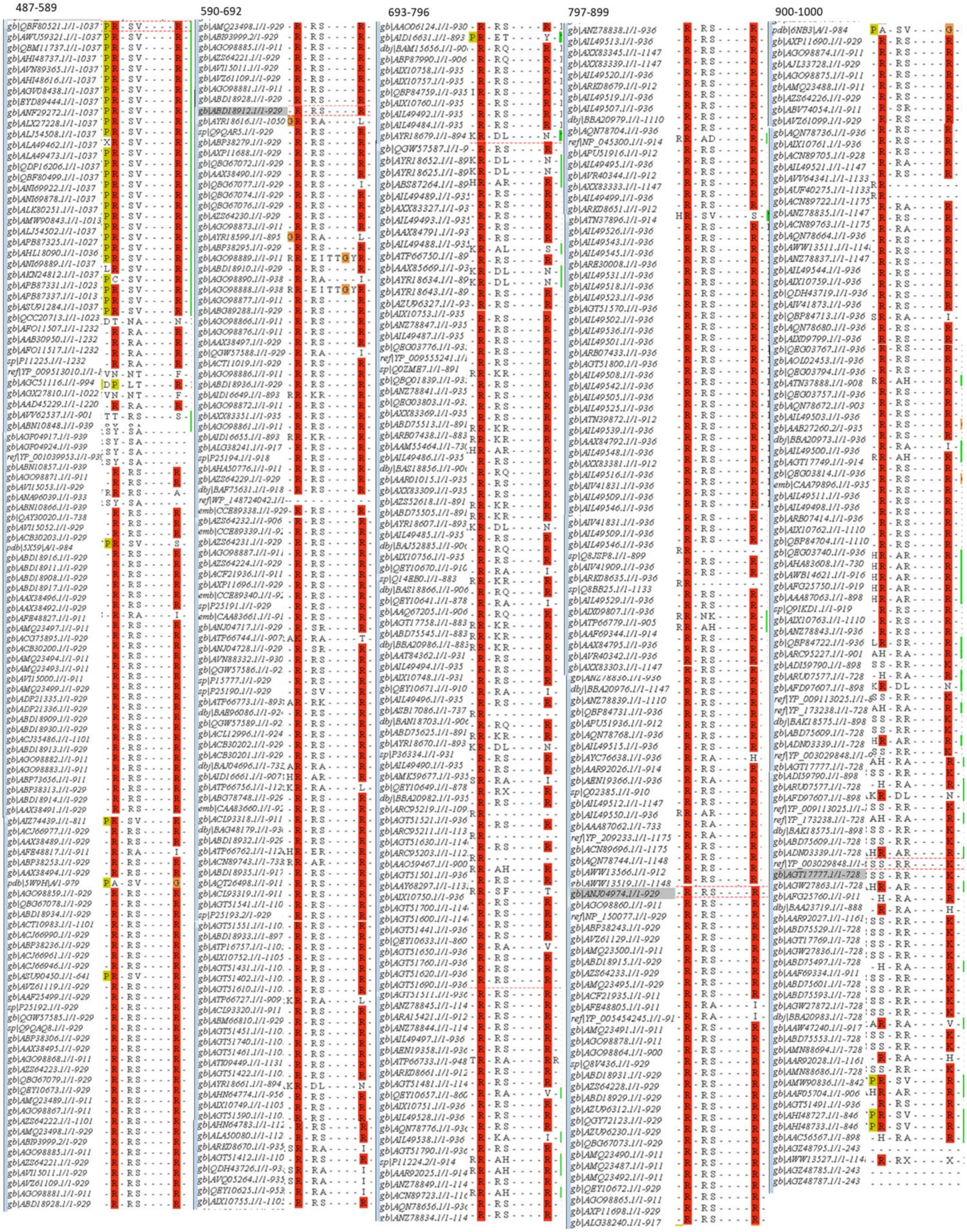

Figure S2. Multiple sequence alignment of 1000 Spike proteins(487-1000). Related to Figure 1.

Sequence	457	YLYRLFR	KS	0. 179	.
Sequence	458	LYRLFRK	SN	0. 100	.
Sequence	462	FRKSNLK	PF	0. 069	.
Sequence	466	NLKPFER	DI	0. 154	.
Sequence	509	VGYQPYR	VV	0. 091	.
Sequence	528	ATVCGPK	KS	0. 059	.
Sequence	529	TVCGPKK	ST	0. 146	.
Sequence	535	KSTNLVK	NK	0. 066	.
Sequence	537	TNLVKNK	CV	0. 093	.
Sequence	557	VLTESNK	KF	0. 057	.
Sequence	558	LTESNKK	FL	0. 091	.
Sequence	567	PFQQFGR	DI	0. 127	.
Sequence	577	DTTDAVR	DP	0. 076	.
Sequence	634	QLTPTWR	VY	0. 098	.
Sequence	646	SNVFQTR	AG	0. 103	.
Sequence	682	TQTNSPR	RA	0. 111	.
Sequence	683	QTNSPRR	AR	0. 146	.
Sequence	685	NSPRRAR	SV	0. 620	*ProP*
Sequence	733	LPVSMTK	TS	0. 065	.
Sequence	765	FCTQLNR	AL	0. 076	.
Sequence	776	IAVEQDK	NT	0. 071	.
Sequence	786	EVFAQVK	QI	0. 066	.
Sequence	790	QVKQIYK	TP	0. 056	.
Sequence	795	YKTPPIK	DF	0. 079	.
Sequence	811	ILPDPSK	PS	0. 058	.
Sequence	814	DPSKPSK	RS	0. 071	.
Sequence	815	PSKPSKR	SF	0. 333	.
Sequence	825	EDLLFNK	VT	0. 053	.
Sequence	835	ADAGFIK	QY	0. 094	.
Sequence	847	LGDIAAR	DL	0. 105	.
Sequence	854	DLICAQK	FN	0. 064	.
Sequence	905	AMQMAYR	FN	0. 169	.
Sequence	921	VLYENQK	LI	0. 065	.
Sequence	933	FNSAIGK	IQ	0. 067	.
Sequence	947	TASALGK	LQ	0. 077	.
Sequence	964	ALNTLVK	QL	0. 079	.
Sequence	983	LNDILSR	LD	0. 068	.
Sequence	986	ILSRLDK	VE	0. 129	.
Sequence	995	AEVQIDR	LI	0. 081	.

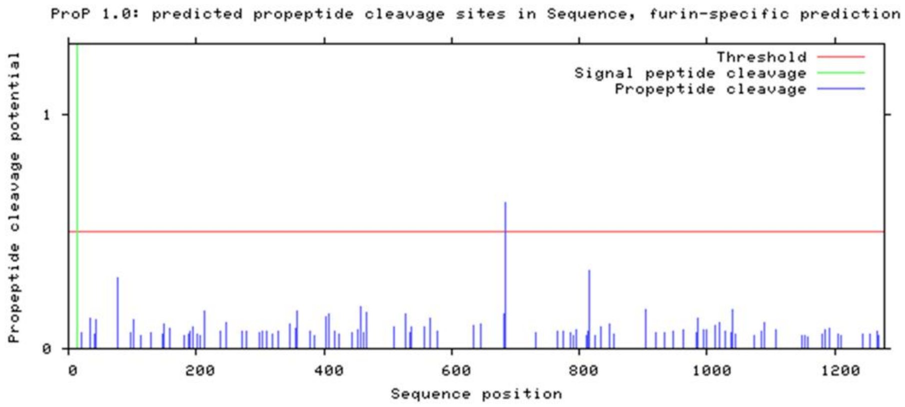

Figure S3. Result of furin cleavage site pridiction of Spike protein in SARS-CoV-2, which predicted by online method ProP 1.0 Server. Related to Table 1.

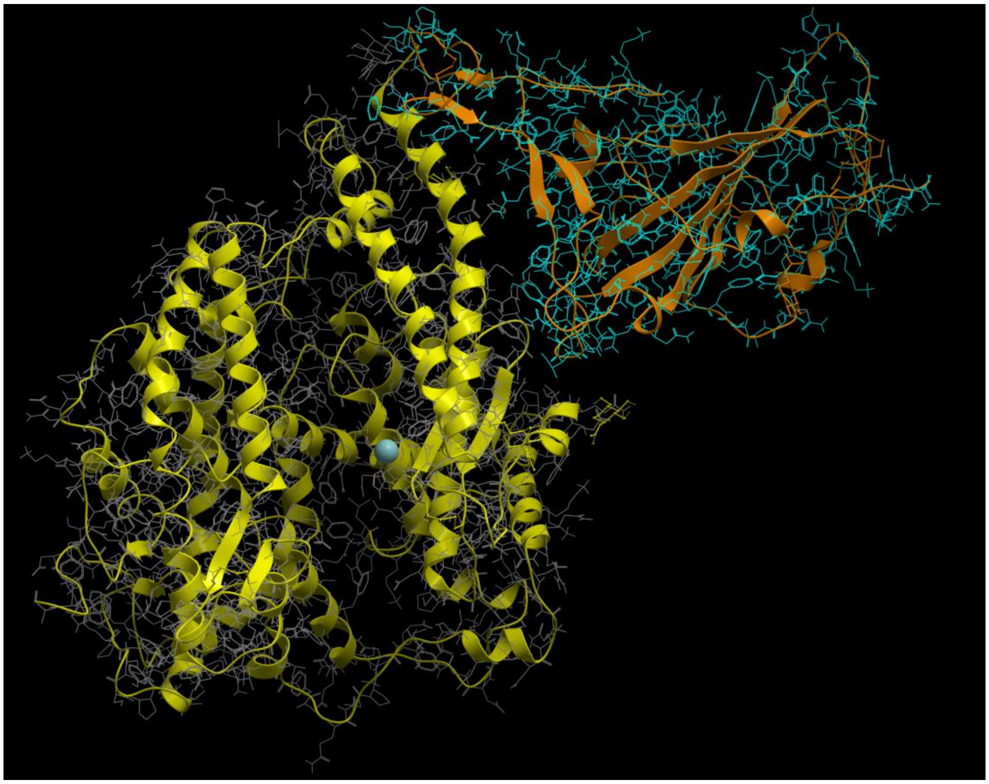

Fig S4 Protein-protein docking calculation model of SARS-CoV-2 spike RBD (light blue) with human ACE2 (yellow), original RBD conformation was shown in orange. The calculated free energy is -50.13 Kcal/mol. Related to Figure 3.

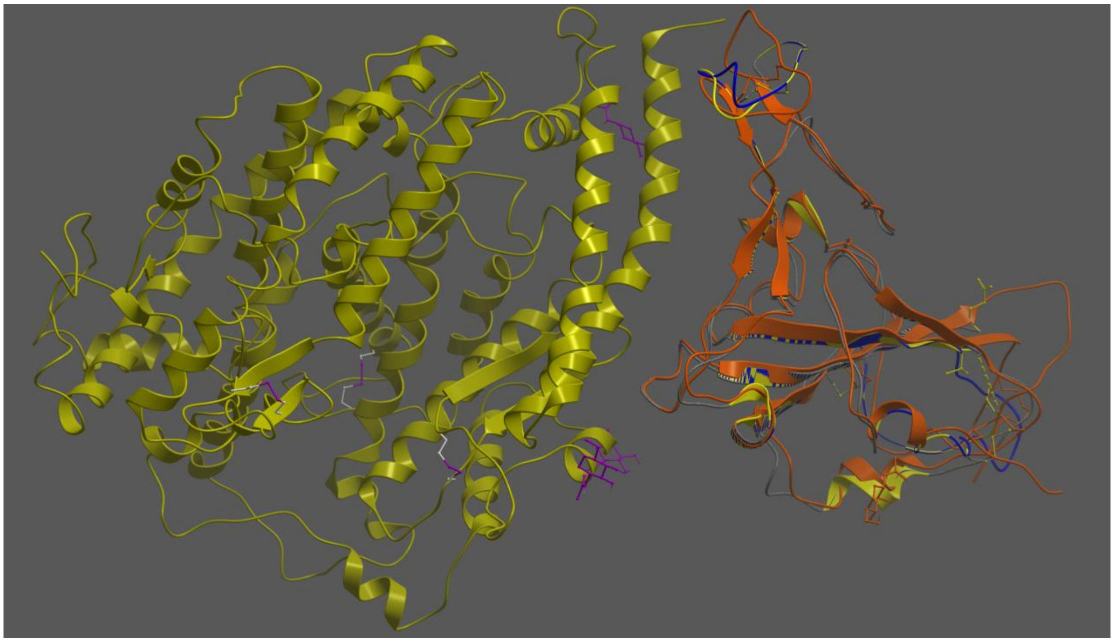

Fig S5 Comparison of SARS-CoV-2 spike RBD (orange) and SARS spike RBD (yellow). The complex with ACE2 (left part, yellow) was shown. The homology model of SARS-CoV-2 spike RBD built from SARS spike RBD was shown as blue. Related to Figure 3.

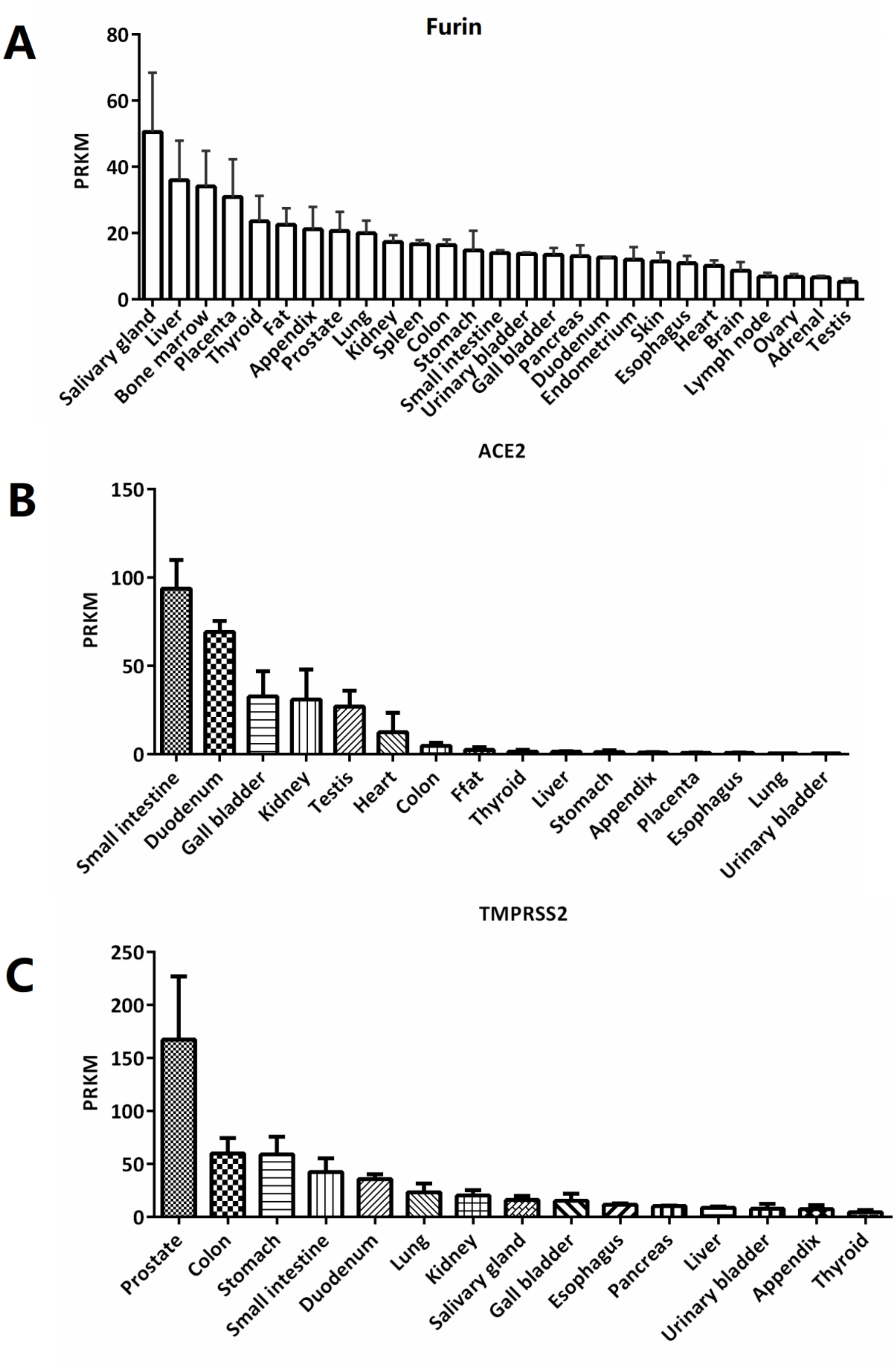

Figure S6. Expression levels of Furin, ACE2 and TMPRSS2 in various tissues. The data is from pubmed. Related to Figure 2B.

Table S1 Potential furin inhibitors from ZINC drug database, related to Figure 4

No.	Drug Name	Structure	Pharmacological functions
1	Aminopterin	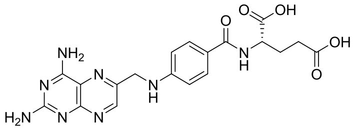	Anti-tumor
2	Folic acid	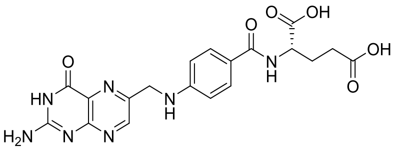	Vitamin B9, necessary material for the growth and reproduction of body cells
3	Sulfoxone	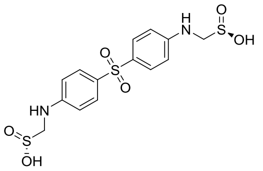	Antibacterial effect
4	Silybin	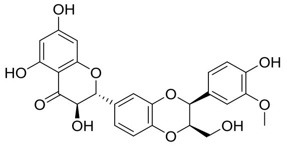	Hepatoprotective effect
5	Diminazene	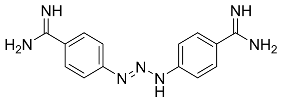	Insecticidal effect
6	Fludarabine phosphate	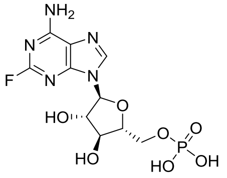	Anti-tumor
7	L-Arginine	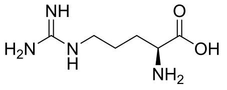	Nutritional supplement
8	Hydroxystilbamidine	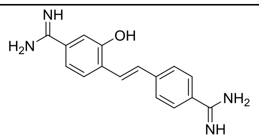	Antifungal effect
9	Methotrexate	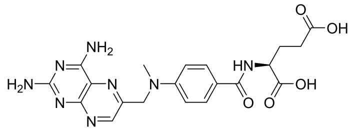	Antineoplastic, antirheumatic effects

10	L-dopa	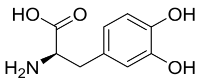	Treatment of Parkinson's disease
11	Irinotecan	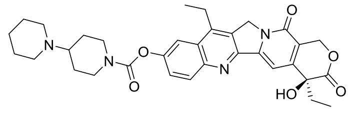	Anti-tumor
12	Cefoperazone	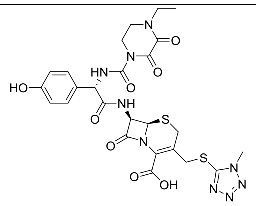	Antibacterial effect
13	Folinic acid	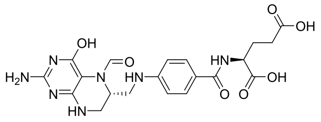	Folic acid supplement
14	Glycerol 3-phosphate	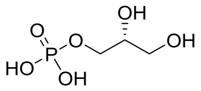	Intermediate for serine synthesis
15	Valganciclovir	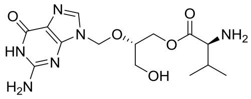	Antivirus
16	Fosaprepitant	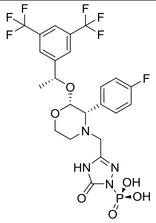	Treatment of nausea and vomiting induced by chemotherapy
17	Lomefloxacin	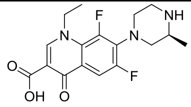	Antibacterial effect
18	Glutathione	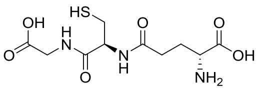	Hepatoprotective effect
19	Famotidine	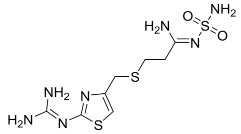	Treatment of gastrohelcosis
20	Imatinib	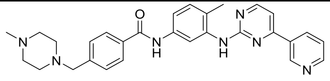	Anti-tumor
21	Chenodeoxycholic acid	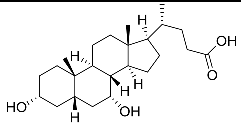	Dissolving gallstones

Table S2 Potential furin inhibitors from in-house natural product database, related to Figure 5

No.	Drug Name	Structure	Pharmacological functions	Source
1	(-)-Epigallocatechin gallate	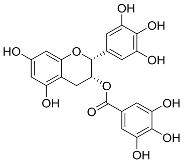	Antioxidation, anti-tumor, treatment of depression	*Camellia sinensis*
2	Theaflavin 3,3'-di-*O*-gallate	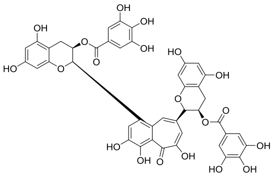	Antioxidant effect, anti-tumor, anti-virus	*Camellia sinensis*
3	Biorobin	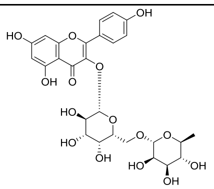	Anti-virus	*Ficus benjamina*
4	14-deoxy-11,12-didehydroandrographiside	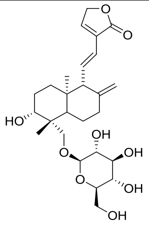	Anti-virus, anti-inflammatory effect	*Andrographis paniculata*
5	(1*S*,2*R*,4a*S*,5*R*,8a*S*)-1-formamido-1,4a-dimethyl-6-methylene-5-((*E*)-2-(2-oxo-2,5- dihydrofuran-3-yl)ethenyl) decahydronaphthalen-2-yl 5-((*R*)-1,2-dithiolan-3-yl) pentanoate	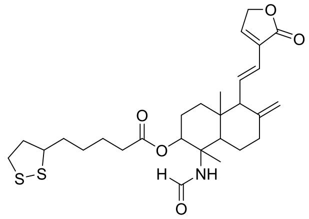	Anti-virus, anti-inflammatory effect	Andrographolide derivatives
6	2*β*,30*β*-dihydroxy-3,4-seco-friedelolactone-27-lactone	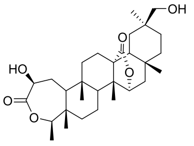	Anti-virus	*Viola diffusa*

7	Phyllaemblicin G7	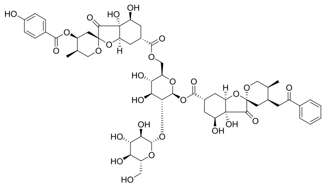	Anti-virus	*Phyllanthus emblica*
8	Andrographolide	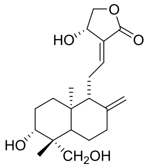	Anti-virus, anti-inflammatory effect	*Andrographis paniculata*
9	14-deoxy-11,12-didehydroandrographolide	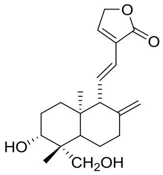	Anti-virus, anti-inflammatory effect	*Andrographis paniculata*
10	(1*S*,2*R*,4a*S*,5*R*,8a*S*)-1-formamido-1,4a-dimethyl-6-methylene-5-((*E*)-2-(2-oxo-2,5-dihydrofuran-3-yl)ethenyl) decahydronaphthalen-2-yl 2-aminoacetate	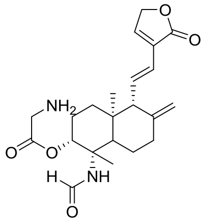	Anti-virus, anti-inflammatory effect	Andrographolide derivatives
11	2-[[2-*O*-(6-deoxy-*α*-L-mannopyranosyl)-*β*-D-xylopyranosyl]oxy]-1,8-dihydroxy-6-ethoxy-9*H*-xanthen-9-one	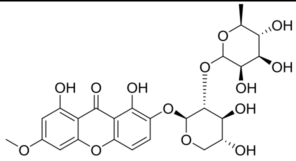	Anti-virus, anti-inflammatory effect	*Swertia kouitchensis*
12	Kouitchenside J	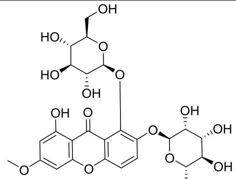	Anti-virus, anti-inflammatory effect	*Swertia kouitchensis*
13	Stigmast-5-en-3-ol	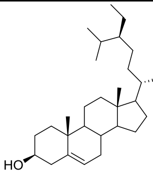	Antioxidant effect	*Spatholobus suberectus dunn*
14	Kouitchenside F	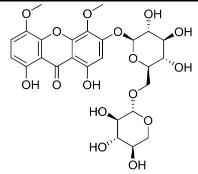	Anti-virus, anti-inflammatory effect	*Swertia kouitchensis*

Table S3

Potential furin inhibitors from the common antiviral drugs database, related to Figure 6

No.	Drug Name	Structure	Pharmacological functions
1	Suramin	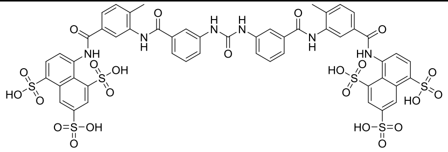	DNA topoisomerase II inhibitor
2	Indinavir	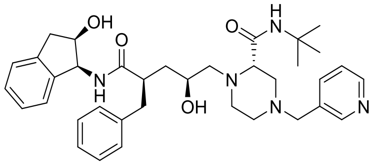	Human immunodeficiency virus Protease (HIV PR), anti-malaria
3	Boceprevir	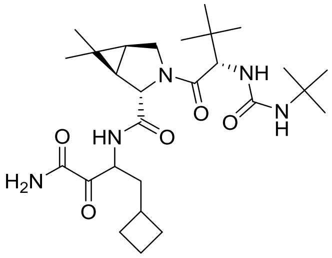	Hepatitis C virus Serine protease NS3/4A (HCV NS3/4A) Modulator
4	Tenofovir alafenamide	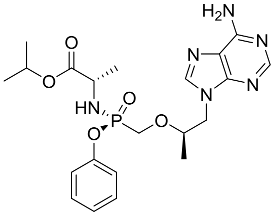	HIV-1, HBV nucleotide reverse transcriptase inhibitor
5	Tenofovir disoproxil	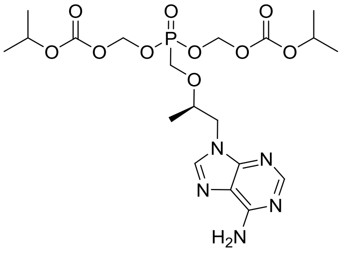	HIV, HBV nucleotide reverse transcriptase inhibitor
6	Acycloguanosine triphosphate	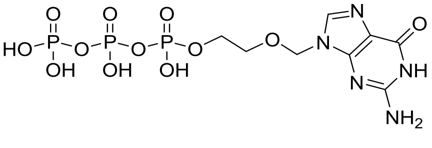	Thymidine kinase of herpesvirus

7	Telaprevir	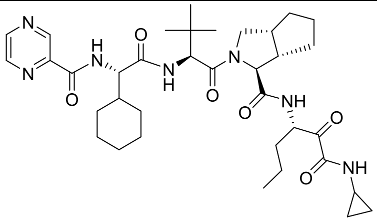	Hepatitis C virus Serine protease NS3/4A (HCV NS3/4A) Modulator
8	Dolutegravir	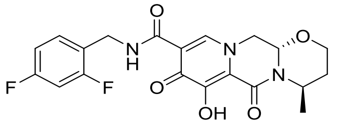	Human immunodeficiency virus Integrase (HIV IN)
9	Maraviroc	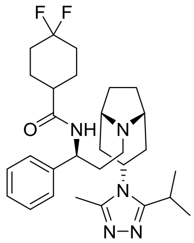	1.C-C chemokine receptor type 5 (CCR5)<br>2.CCR5 messenger RNA(CCR5 mRNA)
10	Cobicistat	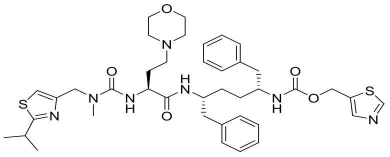	Inhibitor of cytochrome P450 3A (CYP3A) enzymes
11	Stavudine triphosphate	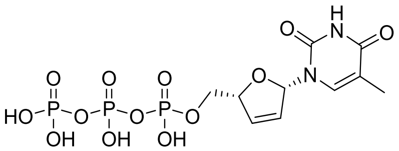	Nucleoside analogue reverse transcriptase inhibitor used in the treatment of HIV infection

Table S4. In vitro furin inhibitory effects of screening hits. Related to Figure 7.

Compounds name	In vitro furin protease inhibition percentage at 100μM(％)	IC50 (μM）
Aminopterin	72	>30
Folic acid	<30	>30
Sulfoxone	<30	>30
Silybin	74	>30
Diminazene	95	5.42 ±0.11
Fludarabine phosphate	<30	>30
L-Arginine	<30	>30
Hydroxystilbamidine	<30	>30
Methotrexate	73	>30
L-dopa	<30	>30
Irinotecan	<30	>30
Cefoperazone	<30	>30
Folinic acid	44	>30
Glycerol 3-phosphate	<30	>30
Valganciclovir	<30	>30
Fosaprepitant	<30	>30
Lomefloxacin	40	>30
Glutathione	<30	>30
Famotidine	<30	>30
Imatinib	58	>30
Chenodeoxycholic acid	<30	>30
Suramin	<30	>30
Indinavir	<30	>30
Boceprevir	<30	>30
Tenofovir alafenamide	<30	>30
Tenofovir disoproxil	38	>30
Acycloguanosine triphosphate	<30	>30
Telaprevir	<30	>30
Dolutegravir	<30	>30
Maraviroc	<30	>30
Cobicistat	<30	>30
Stavudine triphosphate	<30	>30
(-)-Epigallocatechin gallate	<30	>30

Theaflavin 3,3'-di-O-gallate	<30	>30
Biorobin	<30	>30
14-deoxy-11,12-didehydroandrographiside	<30	>30
Phyllaemblicin G7	<30	>30
Andrographolide	<30	>30
14-deoxy-11,12-didehydroandro grapholide	<30	>30
Kouitchenside J	<30	>30
Stigmast-5-en-3-ol	<30	>30
Kouitchenside F	<30	>30
